# The implementation of smart continence care for people with disabilities: A qualitative study of key stakeholders’ first-hand experiences

**DOI:** 10.1177/20552076241290399

**Published:** 2024-10-15

**Authors:** Julia FE van Calis, Vivette JC van Cooten, Odile Smeets, Jenneken Naaldenberg, Brigitte Boon, Geraline L Leusink, Kirsten E Bevelander

**Affiliations:** 1 Department of Primary and Community Care, 6034 Radboud University Medical Center, Nijmegen, The Netherlands; 2 560794 Academy Het Dorp, Research & Advisory on Technology in Long-term Care, Arnhem, The Netherlands; 3 Tranzo, 200730 Tilburg School of Social and Behavioral Sciences, Tilburg University, Tilburg, The Netherlands; 4 Siza, Center for Long-term Care for People with Disabilities, Arnhem, The Netherlands

**Keywords:** Continence care, care technologies, implementation, qualitative study, experiences, disability care, person-centred care, eHealth

## Abstract

**Introduction:**

Innovative technological applications like smart continence care (SCC) offer potential benefits in healthcare delivery, particularly for individuals with profound intellectual and multiple disabilities (PIMD). SCC aims to prompt caregivers to change continence materials, potentially improving clients’ quality of life and reducing caregivers’ workload. Although the use of SCC in PIMD care is promising, research is needed to improve its use in these complex settings. The aim of this study was to reflect on the SCC implementation process in care organizations for people with PIMD.

**Methods:**

Fifteen semi-structured interviews were conducted with key stakeholders, caregivers, and SCC project leaders, across four care organizations. Interviews utilized an integrated framework drawing from the centre for eHealth research roadmap and the nonadoption, abandonment, scale-up, spread, and sustainability framework, both addressing eHealth implementation. Thematic analysis and open coding were employed to identify key themes and sub-themes in the implementation process.

**Results:**

Four main themes emerged as crucial for successful SCC implementation: creating support, communication between stakeholders, problem-solving, and willingness to adopt SCC. The first three themes were perceived as contributors to the success of SCC implementation, whereas the last theme showed factors impacting willingness to adopt SCC. Early involvement of key stakeholders and clear communication about expectations of their roles was perceived as crucial and created clarity. Adequate problem-solving was identified as influential in SCC utilization and willingness to adopt person-centred continence care.

**Conclusion:**

Implementation of SCC requires changes in the work routines of those involved, and key stakeholders’ early involvement appears to improve support for these changes. Fostering communication between key stakeholders and adequate problem-solving contributes to positive experiences and the perceived success of sustainable implementation. This study offered a rich understanding of day-to-day practices around implementing SCC in disability care organizations. The findings may also be relevant for the implementation of technologies in other care settings.

## Background

Incontinence is common among people with profound intellectual and multiple disabilities (PIMD).^1–4^ Most people with PIMD cannot notify professional caregivers in residential care (hereafter referred to as caregivers) when their incontinence material needs to be changed because of their limited communication, cognitive, and motor skills.^1–4^ This leads to a highly intensive care process.^5,6^ Advanced technologies, such as the ones used for continence materials for smart continence care (SCC), can help people with PIMD to notify their caregivers when their continence material has reached a certain saturation level and needs to be changed.^7^

The use of technological applications, such as for SCC, in (health)care settings may contribute to the improvement of the quality of care and person-centred care.^8,9^ The implementation in daily care is challenging and complex considering the magnitude and the heterogeneity of the disabilities among people with PIMD.^10^ Therefore, caregivers need to be aware of the specificity of the disabilities and needs of each individual.^5,10^ However, usually, caregivers change incontinence materials at fixed times. Providing continence care at the time when incontinence material is saturated prevents leakages and avoids unnecessary changes of incontinence material, which leads to more sustainable and personalized care.^11^ This is predicted to enhance clients’ quality of life while reducing the workload for caregivers.^7^ Previous studies have demonstrated the applicability and effectiveness of various SCC systems in elder care settings.^11–13^ Despite its potential to enhance the care for people with PIMD,^7^ experiences with SCC implementation in disability care organizations have not yet been structurally investigated.

Numerous theoretical frameworks exist on technology implementation.^14^ More general frameworks are, for example, the updated Consolidated Framework for Implementation Research (CFIR),^15^ Wensing and Grol's work,^16^ or diffusion of innovation in service organizations.^17^ Additionally, there exist eHealth frameworks specifically tailored to implementation processes of healthcare technologies,^18^ such as the centre for eHealth research (CeHRes) roadmap and the nonadoption, abandonment, scale-up, spread, and sustainability (NASSS) framework. In the current study, we reflected on the SCC implementation process by using an integrated framework combining elements from the CeHRes roadmap and NASSS^19^ to ensure a comprehensive understanding of the complexities involved within the context of implementing healthcare technologies.

The CeHRes roadmap and NASSS have an empirical base in healthcare technology research.^20–23^ The CeHRes roadmap can be applied to guide and reflect on the development, implementation, and evaluation of eHealth technologies.^24,25^ The NASSS framework guides and reviews the implementation of health and care technology in multiple domains (e.g., technology, value proposition, adopters, organization).^26^ According to NASSS, technology development is a never-ending process in which technology can be adjusted to fit each specific setting and context, showing important preconditions for implementation.^27^ The two frameworks complement each other and provide a comprehensive and iterative perspective on the complexity of the implementation process in healthcare and can be applied to guide and reflect on this process.^25^

The main objective of this study was to reflect on the SCC implementation process in disability care organizations for people with PIMD by applying the integrated implementation framework^19^ in gathering first-hand experiences from key stakeholders, i.e., caregivers and project leaders directly involved in applying SCC.

## Methods

### Study design

This study used a qualitative design with semi-structured interviews to gather the experiences of key stakeholders from four disability care organizations involved in SCC implementation. The COREQ checklist was used as a reporting guideline (see Supplementary File 1). The participants gave their informed consent for their interview and recording. Ethical approval was obtained from the Medical Ethics Committee of Radboudumc (NL72751.091.20).

### Study setting

This study was part of a 3-year cluster randomized trial aimed at researching the (cost-)effectiveness of SCC for people with PIMD.^7^ Six disability care organizations participated in the trial, of which four were invited to participate in the present study because of the stage of the implementation at the time. Each of these care organizations implemented SCC for 26–31 persons with PIMD, distributed over four to seven residential homes. All these care organizations set up a project team and appointed a project leader specifically for SCC implementation. An implementation guideline, which will be published in due course, was developed a priori based on implementation theories and evidence^15,16,28–30^ and refined through co-creation with the participating care organizations. Additional consultation was available during the implementation process. The SCC used in this study consists of diapers and pads that contain urine sensors.^a^ A removable clip attached to the incontinence material sends information about the saturation level to a receiver via Bluetooth and subsequently to an app on the caregiver's mobile phone. Caregivers receive colour-coded notifications depending on the set saturation level per user: change desired (orange), risk of leakage (red), or OK (green).

### Participants

Purposive sampling was used to include a broad diversity of the experiences of the key stakeholders responsible for coordinating and executing SCC implementation in the four participating care organizations. The researchers involved in the implementation process identified key stakeholders who were closely involved in the implementation process of SCC. Of the 16 invited key stakeholders, 15 participated (14 females, 1 male). The study sample consisted of project leaders (n = 8) involved in the implementation process at these four organizations and caregivers (n = 7). The project leader group consisted of four participants specifically appointed to lead SCC implementation in their organization. Five participants were closely involved because of their daily work, two as team coordinators, one project coordinator, one programme manager, and a person combining the role of team manager and project leader. The caregivers interviewed were appointed or volunteered as SCC ambassadors, meaning that they were responsible for implementing SCC in their team and serving as the key SCC user. They were the spokesperson to the project leader and the SCC supplier. We, therefore, refer to the caregivers with an ambassador role group as ‘ambassadors’ when they spoke about their role as ambassador. We refer to them as caregivers when they spoke about their own role as professional caregivers, or their team members’ caregiver role. Since this interview was part of the trial they were already participating in, all interviewees were informed beforehand that they would receive an invitation for an interview.

### Study procedure

Interviews were conducted between January 2022 and December 2022 by JvC and VvC. The duration of the interviews varied between 30 and 60 min. Twelve interviews were online, using Microsoft Teams; the other interviews were conducted onsite at a location of the interviewees’ choice. The interviews were transcribed, and participants were offered a member check, which did not lead to any changes.

The interviews were tailored to match project leaders, caregivers, and their specific organizations based on the implementation logbook, kept by VvC, which contained information about salient events. This resulted in two topic lists developed based on the integration of the CeHRes roadmap and NASSS framework.^19^ The topic lists incorporated nine domains derived from this integrated framework: (1) participatory development, (2) iterative process, (3) value specification, (4) value proposition, (5) technological development and design, (6) organization, (7) external context, (8) implementation, and (9) evaluation.^19^ Tables 1 and 2 give an overview of how the topics, interview questions, and domains relate to one another. The first interview with a project leader served as a pilot, after which minor adjustments were made.

**Table 1.** Topic guide interviews: project leaders.

Interview topics	Example questions	Domains^19^ (van Calis et al., 2023)
1. Stakeholders	Who are the stakeholders in this project and how did they become involved?	Participatory design/research
	How have the expectations of the various stakeholders been dealt with?	Value specification
2. Locations	What changes in routines were needed to implement SCC?	Implementation
3. (Technical) Implementation	What did the technical implementation of SCC within the organization and participating locations look like?	Technological development and design
		Organization
	Were any problems encountered?	Implementation
4. Iterative process	Was there room for feedback and evaluation during the implementation?	Iterative process
5. Upscaling	What is your view towards upscaling smart continence care within your organization?	Organization
	Who had ownership during the project?	Value proposition
6. Lessons learned	What is the most important lesson that you learned?	All domains represented
7. Evaluation	What is the impact of SCC on the users (clients and healthcare providers) according to the interviewees?	Evaluation

**Table 2.** Topic guide interviews: ambassadors.

Interview topics	Example questions	Domains^19^ (van Calis et al., 2023)
1. Usage of innovation/SCC	What are the experiences of you and your colleagues with using SCC?	Implementation
2. Implementation process	How do you fulfil the role of ambassador?	Implementation
		Iterative process
3. Impact of SCC	What added value did SCC give?	Evaluation
4. Upscaling	Would you like to continue using SCC?	Organization
		Value proposition
5. Evaluation	What are your suggestions for implementing SCC within other teams?	Evaluation

### Data analysis

After transcription, the qualitative data were analysed thematically, supported by the ATLAS.ti 9.1.6 software, in several steps, as described by Braun and Clarke.^31,32^ To gain familiarity with the data, transcripts were read, and audio recordings were listened to. The frameworks (CeHRes and NASSS) were used to reflect on the implementation process. We applied open coding to the data and analysed the data inductively, with the aim of formulating new themes based on the data itself rather than assessing the existing frameworks. Two authors (JvC, VvC) separately open-coded three transcripts. Relevant quotes and codes were identified and similarities and differences in coding were discussed, resulting in a first conceptual codebook. This codebook was subsequently applied to six transcripts independently by the two main authors (JvC, VvC). Discussing and comparing the codes between three authors (JvC, VvC, KB) led to the addition of new codes, rephrasing codes, and merging codes, resulting in a coding structure. This coding structure was applied to the remaining transcripts by two authors (JvC, VvC).

Next, two authors (JvC, VvC) discussed all coded data. In several rounds, codes were clustered into broader overarching categories. A third author (KB) joined in the last rounds of this step, leading to a final review and (re)naming the main themes and sub-themes. For example, the codes ‘discuss’, ‘inform’, ‘evaluate’, and ‘advise’ were clustered into the sub-theme ‘continuous communication’ as part of the main theme ‘communication between stakeholders’. Three authors (JvC, VvC, KB) grouped the sub-themes, resulting in four main themes described in the results section below.

## Results

The analysis resulted in four main themes: (1) create support, (2) communication between stakeholders, (3) problem-solving, and (4) willingness to adopt SCC. Each theme has two or three sub-themes, which are described in this section and illustrated by quotes in the tables. The first three themes can be defined as contributors to the success of the SCC implementation, whereas the last theme shows how these factors impact the willingness to adopt SCC.

### Create support

Support for SCC use was created through engagement during implementation itself and included several roles that key stakeholders played in the process (Table 3).

**Table 3.** Sub-themes and quotes of the theme create support.

Sub-themes	Quote-reference	Quotes
Engagement during implementation	1.1.a	‘Then, I look for the account manager [of the IT department] at the different regions within our organization. Then, when I knew this, I involved them. So that they knew what was going to happen. Next, they looked for IT persons who can provide support on-site. So, when there were questions, this was the person to go to. So, in all, that's how we involved them’ (Project coordinator, organization 4).
	1.1.b	‘I appreciate the fact that they informed me before starting to use the smart diaper. That's good, (…) because, often they forget the night team in such a project’ (Ambassador and night care coordinator, organization 1).
Roles in the implementation process	1.2.a	‘The guidance [of the project leader and program manager] was good. They even visited our location. They were happy to give an explanation and we discussed how things can be put to practice for the night care team’ (Ambassador and night care coordinator, organization 1).
	1.2.b	‘I haven’t even spoken with my manager. Therefore, all the problems we encountered are still there’ (Ambassador and caregiver, organization 4).
	1.2.c	‘Ambassadors get hours for this project, like 4 h per week. If they need more, they should tell me. I don’t check the hours as a program manager, but I do report these hours at the end of each quarter of a year for the administration and internal budgeting’ (Program manager, organization 1).
	1.2.d	‘Yes, however, I must admit that some colleagues changed diapers out of precautions, although we did not receive a notification on the phone. They were actually scared that if they waited, then within one hour there would be a notification, and then it would not be a good moment. So, yes, that is not the way we are supposed to act. But, yeah, it just happens out of precautions, because then you think, “I don’t have to change diapers at 15 h of 16 h”’ (Ambassador and caregiver 1, organization 3).

#### Engagement during implementation

Early in the implementation process, stakeholders were engaged consciously by the project leaders. This was perceived as having a positive influence on creating support for SCC implementation by the caregivers. For instance, the early involvement of support services such as IT and logistics departments was important, because they provided essential preconditions to contribute to the implementation, and by early involvement they were able to think along and prepare the implementation from their side [Quote 1.1.a]. Early involvement of managers is important to support SCC use in the organization and to ensure joint decision-making for possible further uptake.

Participants stated that the SCC implementation meant a change in work routine, from changing diapers at fixed times to diaper changing when a notification indicated full incontinence material. Caregivers felt supported when they were informed about the project before the implementation started [Quote 1.1.b]. Because of this early involvement, the expected added value of SCC for the client and the workplace was more evident to those involved; this was experienced as motivating and increased willingness to cooperate. Caregivers described the expected added value as the increased well-being of the clients with PIMD, elimination of unnecessary material changes, improved skin condition, and learning about clients’ voiding patterns.

#### Roles in the implementation process

Project leaders had a managing role in the implementation process, entailing the management of implementation at participating locations and keeping all stakeholders involved and connected by coordinating the necessary implementation steps and communicating with management and the SCC supplier. In this role, project leaders also had to deal with resistance from the caregivers, for instance, regarding diaper-changing routines. Therefore, proximity of the project leaders and ambassadors to first-hand users on the work floor – the caregivers – was considered important and led to positive experiences [Quote 1.2.a]. In line with this, a lack of contact and proximity resulted in negative experiences [Quote 1.2.b].

Caregivers who were ambassadors in the implementation process acted as key users to their colleagues. In this role, they were the liaison between the project leader, their team, and the supplier. This role was new to them and included tasks to involve and support colleagues in using SCC, changing work routines, and signalling issues as well as communicating with the project leader and the SCC supplier. Allocating dedicated hours and being explicitly assigned the role of ambassador was an important facilitator [Quote 1.2.c], as were their skills in dealing with resistance. Ensuring a collaborative feeling through close contact between the teams, ambassador, and project leaders created a feeling of ‘doing it together’, thereby contributing to a positive experience.

SCC implementation and use eventually takes place on the work floor. It is the caregivers that need to change their way of working. Although the caregivers stated that they understand the potential added value of SCC and understand that the existing routine is not optimal, it still can be hard for them to let go of the fixed schedules [Quote 1.2.d] and change on request indicated by the SCC has an impact on the rest of the day's programme.

### Communication between stakeholders

Communication between different stakeholders entailed two sub-themes: continuous communication and communication channels (Table 4). Communication took place with external stakeholders, such as the supplier of the SCC material, but also between internal stakeholders, such as different care teams, the project leader, support services, and management of the care organization.

**Table 4.** Sub-themes and quotes of the theme communication between stakeholders.

Sub-themes of communication between stakeholders	Quote-reference	Quotes
Continuous communication	2.1.a	‘We had our team meetings, in which we evaluated. And the “[person] from IT or innovation” came by every week to evaluate. And, if I was downstairs, the locations are downstairs, if I was at the residence, I always asked them “how is it going?” “how is it?” These were the informal moments which were highly informative’ (Team manager and project leader, organization 4).
	2.1.b	‘It felt like we were muddling along. Things were discussed during that meeting [2 months ago]. But the [person] from innovation didn’t do anything with this. And the manager neither, I haven’t even spoken with my manager. Therefore, all the problems we encountered are still there’ (Ambassador and caregiver, organization 4).
Communication channels	2.2.a	‘In my opinion, the communication is handled in very different ways. There are caregivers who are in panic and frustrated [as the SCC was not working properly]. They send not-so-nice WhatsApp messages [to the supplier of SCC] and are not happy if they don’t get a response within 20 s. On the other hand, having a message in text can be tricky as well’ (Team coordinator 1, organization 3).
	2.2.b	‘[The product specialist of the supplier] had a few locations he preferred, and therefore spent more time on these locations. Other locations hardly got any attention’ [irritated tone of voice] (Project leader, organization 2)

#### Continuous communication

Continuous communication gave the participants the experience of support and direction in what was needed for implementing SCC at multiple stages of the implementation process. During the preparation phase, this included informing many people throughout the organization and asking for their advice, for example, about the size of material to use and where to place the Bluetooth receivers. During SCC use, the care teams, together with the supplier, often discussed and evaluated SCC per individual client, so that adjustments could be made to ensure optimalization for the client. These junctures, together with informal points of contact [Quote 2.1.a], ensured continuous communication. This was an important prerequisite to keep the implementation on track and prevent delays arising from unsolved problems and inaction, which could result in a negative experience of ‘muddling through’ [Quote 2.1.b].

#### Communication channels

The continuity of communication as described above was facilitated in different ways and through various channels, such as email, WhatsApp, phone, online meetings, and live meetings. Both online and offline meetings between care teams from different locations were scheduled, thereby ensuring that everyone could be involved in the changes that took place and in the agreements that they made with one another.

Caregivers experienced WhatsApp as a fast, accessible, and direct way of communication between the work floor and the supplier. A risk with this type of communication was that expectations were not always met and that misunderstanding could arise as a result of differences in the interpretation of written text in WhatsApp [Quote 2.2.a]. Frequent face-to-face contact was experienced as pleasant and helpful, especially when client-specific cases needed to be discussed. When there was too little face-to-face contact, this led to frustration [Quote 2.2.b].

### Problem-solving

When starting the SCC implementation process, teams encountered problems and challenges relating to working routines, everyday-care situations, and technical or practical problems with the product itself. During the process, different strategies to address challenges and problems were used, as discussed below (Table 5).

**Table 5.** Sub-themes and quotes of the theme problem solving.

Sub-themes of problem solving	Quote-reference	Quotes
Solving problems on the work floor	3.1.a	‘You notice that the perspective of the product specialist [of the supplier] was very different from our perspective about the process of changing diapers and wet diapers. You do notice that in every discipline, and whether you're talking about night care, or someone working during the day, or the product specialist, everyone has their values and norms and has an opinion. Sometimes it was difficult to come to an agreement about what kind of choice we make. Especially at night, do we let someone lie down or do we go with the app? Yes, these are things that I sometimes found difficult to agree upon and to deal with’ (Ambassador and night care coordinator, organization 1).
	3.1.b	‘(…) within the night care team, people dealt differently with the agreements [about SCC during the night] we made. I could send them an email with ‘that is not what we agreed upon, we have not decided yet’, but still they did their own thing. And if that happens repeatedly, well, yeah, that's that I think’ (Ambassador and caregiver 1, organization 1).
	3.1.c	‘One of the persons with PIMD had a severe form of autism. Caregivers noticed that this person found it very difficult to get used to it [the SCC clip and not having continence care at fixed moments during the day]. So, we stopped using SCC, and then he had to adjust again. So, for some persons, this is very burdensome’ (Project leader, organization 2).
Solving problems with the product	3.2.a	‘Well, yes, my experience was pleasant with this. They [supplier] were very willing to help. They always came up with solutions when something was wrong, and they never made things difficult. The corporation was very pleasant, I must say. They kept their promises. We just had a good working relationship with them’ (Team coordinator 2, organization 3).
	3.2.b	‘Contact with the people from IT was not good, very often we got this “easy answer” that “the mobile signal strengths just are not good enough”. We experienced the same with [employee of supplier]. [This person] did follow up our questions, but then, nothing. Most of the problems we experienced with communication were about technical issues’ (Project leader, organization 1).
	3.2.c	‘Some locations struggled to work according to the notification of the app and wait with changing the diaper. Sometimes, notifications were incorrect, decreasing the trust’ (Project leader, organization 1).
	3.2.d	‘The technical adjustments that need to be made: less incorrect and defect notifications, otherwise we will not implement it. Because, then you need somebody to fix these problems, making the business case more difficult. We discussed this with the supplier and wrote this down in the advisory report which we will discuss internally’ (Program manager, organization 1).

#### Solving problems on the work floor

The situation occurred that one team's caregivers provided continence care in a way that the other team viewed as inappropriate. This arose because of differences in the vision on quality of care and that sleep is more important than changing continence materials. For example, night-care teams contended that the night was for sleep and that their clients should not be woken up for a diaper change, even if this could result in a leakage. Day-care teams, however, felt that waking a person at night was preferred to decrease the possibility of them having a wet bed. It could be difficult to overcome these differences [Quote 3.1.a]; participants felt that it was important to communicate and agree upon how care is delivered; however, this did not always happen [Quote 3.1.b]. In general, the most important thing to do according to the participants is to communicate and explain each other's point of view and together agree upon what is best for the specific client and then keep checking whether the change in continence care provided has the desired effect.

Caregivers encountered several problems in matching the SCC material with their clients’ individual characteristics. This included finding a comfortable size and absorption capacity, determining the notification saturation level that suited the person's skin condition, and determining the right time for a diaper change in line with the client's programme of daily activities. When information about an individual voiding pattern was made available through the online portal, this helped caregivers to determine a care strategy per person. Caregivers could even decide to stop using SCC for some persons with PIMD to match their needs [Quote 3.1.c].

#### Solving problems with the product

Problems relating to technical and practical issues with the SCC product, if not possible for the caregivers or the care organization's IT department to solve, were solved by the supplier. One such problem that appeared, for example, was malfunctions with the Bluetooth clips that either did not work or did not send information to caregivers adequately; this needed to be solved by changing the clip or installing additional receivers. Often, this and other problems were adequately and promptly addressed by the supplier, thereby helping the working relationship between the supplier and caregivers and the caregivers’ work [Quote 3.2.a]. When there were delays in responses, unhelpful solutions, or differences in perceptions of how a problem had to be addressed, this led to frustrations with the caregivers and ambassadors [Quote 3.2.b,] and affected the trust in the product [Quote 3.2.c]. Project leaders indicated that the way in which technical problems were addressed during implementation influenced the decision on further SCC uptake within the organization [Quote 3.2.d].

### Willingness to adopt SCC

Willingness to adopt SCC was influenced by three key factors: product usability, care teams’ attitude and motivation, and the skills and competencies available within the care teams (Table 6).

**Table 6.** Sub-themes and quotes of theme product usability.

Sub-themes	Quote-reference	Quotes
Product usability	4.1.a	‘I didn’t like this product [diaper of the SCC system] as much as the products we used before and using again now. In my experience, these diapers (…) don’t stick that well [the closing mechanism of the diaper]. And I feel that, even the heavy diapers, don’t have that much absorptive capacity compared to the ones we are used to’ (Ambassador and night care coordinator, organization 1).
	4.1.b	‘Quite often they [people with PIMD] were wet, while the SCC system indicated green [no need for change]’ (Ambassador and caregiver 2, organization 3).
Attitude and motivation to adapt to a new way of working	4.2.a	‘Yes, however, I must admit that some colleagues changed diapers out of precautions, although we did not receive a notification on the phone. They were actually scared that if they waited, then within one hour there would be a notification, and then it would not be a good moment. So, yes, that is not the way we are supposed to act. But, yeah, it just happens out of precautions, because then you think, “I don’t have to change diapers at 15 h of 16h”’ (Ambassador and caregiver 1, organization 3).
	4.2.b	‘In the beginning, in the first three weeks, it was just “How are you doing this? How are you doing this?” We really had to support each other in attaching the clips and cleaning it. “You have to put on the diaper this way”. “Ow! Is that how you do it!”. Being proud when you attached the clip. That was during the first few weeks. […]. We proudly informed each other when we attached the clip the right way. That really gave us the feeling of doing it together’ (Ambassador and caregiver 3, organization 1).
	4.2.c	‘Our fixed staff did show interest in the product and the app [of the smart diaper], however not everybody was positive (…) [there were earlier experiences with the smart diaper a few years ago]. Then people said, “There we go again, again the same project”. They did not like it then, so they already had their assumptions, they had already formed their opinion before we even started. It was hard to change their minds’ (Ambassador and night care coordinator, organization 1).
	4.2.d	‘We changed the diaper of the client 4 times per day, which was our standard. But since we use the smart diaper we now know that we did this at the wrong time. We removed a clean one [diaper] and an hour later, you were lucky [diaper contained urine]… I think that we have learned to see technology as a tool, rather than something which will take our jobs’ (Team coordinator 1, organization 3).
	4.2.e	‘Well in the beginning, before we started using the smart diaper, almost nobody got a new diaper before the night started. We had our fixed round between 18 h and 19 h, when everybody got into their pajamas and got a new diaper. With this [diaper], we put them to bed. In the first week [of the smart diaper] it was wonderful to see the data [generated by the smart diaper]. We came to the conclusion that some people were wet during the night. I have sent an email, so that more people get a new diaper before the night [between 21 h and 22 h]. We benefit from this, as we really have more dry beds, and our clients a better sleep’ (Ambassador and caregiver 1, organization 1).
	4.2.f	‘There is one client, who pees a lot at the beginning of the night, and then nothing the rest of the night. The nightcare team tried to change his diaper a few times, but this put a lot of burden on the client. He did not like this at all. So yeah, then you really put stress on him. It was very stressful for him. (…) He was also wearing a leg splint. (…) So, we choose to leave him in bed with a full diaper’ (Ambassador and caregiver 2, organization 3).
	4.2.g	‘There are clients for whom we agreed upon [new] working arrangements. During our round [in the night], we check the app to see the status of the smart diaper. This we do for clients we already agreed upon for visiting in the night. With the app, we can see if we must do the visit now, or if we wait a bit. So, per residence and per client, we have different arrangements in how we deal with the app’ (Ambassador and night care coordinator, organization 1).
Training and skills	4.3.a	‘I did get extra courses and explanations. But in principle, the whole team is educated [on the use of smart diaper], so in that sense they already have a lot of information. I [as an ambassador] did not have to do much in that sense’ (Ambassador and caregiver 1, organization 3).
	4.3.b	‘The training was lacking a lot, it was incomplete, time was spent on less relevant subjects, leaving no time for things that matter (…). And there were locations [the product specialist] preferred and spent more time on, while other locations did not get any, or less attention’ (Project leader, organization 2).
	4.3.c	‘Yes, the [online portal], was explained and they [supplier] assumed that we used it. However, the caregivers did not use it. That is a missed opportunity. It has been mentioned, and yes, at least in my experience, a password was sent, but it was never checked if the caregivers could use it and wanted to use it. So, a missed opportunity if you ask me. And if it is them [supplier] to blame, or my care teams, well we just leave it at that’ (Team manager and project leader, organization 4).
	4.3.d	‘It helps if you have a certain level of knowledge, (…) some people just handle it [technology] easily, others less. I am lucky with one of my caregivers, he really was “top chef smart diaper”. But he really has the competence to sift through and go above and beyond’ (Team coordinator 1, organization 3).
	4.3.e	‘Ambassadors (…) had a leading role (…). They were selected by the team lead and must be able to motivate. I always select people based on “someone who shows enthusiasm and has enough working hours”. It does not have to be the most technical person, but someone who can motivate the team to change. It is even good if the person is not too technical, because then you can check if everything is explained properly’ (Program manager, organization 1).

#### Product usability

Participants had different experiences regarding the usability of the product. For example, the absorption capacity could be different than their usual product, or characteristics like closing mechanisms were experienced as less favourable [Quote 4.1.a]. This influenced willingness to adopt the product after having experienced it. Technical issues or product malfunctions influencing the quality of care [Quote 4.1.b] had an impact on the trust in the product itself and was an important issue raising doubt about its further adoption.

#### Attitude and motivation to adapt to a new way of working

Teams struggled to adapt to the new working routines, as, with SCC, diapers should be changed promptly, instead of according to a fixed time schedule [Quote 4.2.a]. Adapting to this new work routine also meant that a team had to learn how to use SCC. An ambassador explained that her team learned together and helped one another [Quote 4.2.b]. However, previous negative experiences with SCC by colleagues resulted in a less favourable attitude towards using SCC, decreasing motivation, and making implementation more challenging [Quote 4.2.c].

Caregivers experienced the SCC as a tool to achieve person-centred continence care [Quote 4.2.d]. Considering the personal information about each participant's voiding volumes and patterns, caregivers were rethinking the thickness of the incontinence products used and the time at which they were putting on a diaper for the night [Quote 4.2.e]. Thinking about how to provide continence care in such a way that it best suits the needs of that specific person was a motivator for caregivers to use SCC. However, this also meant that sometimes the best thing to do was not to adhere to the SCC notifications [Quote 4.2.f].

Another example of when care teams did not adhere to the SCC notifications was when they decided to combine changing of continence material with the transfer of the person with PIMD to bed during the day. They explained that, because of the transfer by patient lift, they preferred to change the diaper during this transfer, avoiding an extra burdensome transfer later on. Thus, caregivers carefully considered when to provide continence care, for which SCC provided them with additional information to guide their decision [Quote 4.2.g].

#### Training and skills

Caregivers received training on how to use SCC. This entailed instructions on how to put on the diaper and how to use the mobile app. There was additional training about the web portal. The experiences of the training differed across organizations and even between teams [Quote 4.3.a and b]; the instruction on the web portal provided by a product specialist from the supplier was not always enough for the caregivers to put this knowledge into practice [Quote 4.3.c]. The training, together with skills such as technological capability and interest, the ability to motivate others [Quote 4.3.d and e], and personal motivation contributed to a more positive or negative experience in implementing SCC.

## Discussion

The aim of this study was to reflect on the SCC implementation process in disability care organizations for people with PIMD. Based on the analysis of the interviews, we identified four main themes important to the implementation process: create support, communication between stakeholders, problem-solving, and willingness to adopt SCC. The first three themes related to the success of SCC implementation, whereas the last theme related to SCC's practical use impacting the willingness to adopt it. Our findings showed that it is crucial that key stakeholders are involved early in the implementation process and that communication between all the key stakeholders is important to create clarity about expectations of their roles. In addition, adequate problem-solving is important, as it influences SCC use and thus the willingness to provide person-centred continence care.

In line with other implementation theories,^16,33,34^ our findings showed that creating support for the implementation of technology is important. This can be achieved when project leaders responsible for the implementation process ensure early involvement and close proximity of its end-users and by appointing project leaders and caregivers as ambassadors. The ambassador's role can facilitate the implementation process on the work floor. This role is similar to that of change agents as described by Greenhalgh et al., 2004;^17^ it is suggested that change agents are successful if they are selected on the basis of their credibility with the potential users, develop strong personal relations, communicate users’ needs to the supplier, and empower users to evaluate the innovation in order to decide about further uptake.^17^ Both ambassadors and project leaders do this by creating support at different organizational levels, by engaging with other key stakeholders, and by having an intrinsic motivation to use a new (health)care technology. Implementation requires behavioural change among stakeholders in order to implement technological applications properly. Change agents are therefore needed to create and guarantee this behavioural change. This study added empirical evidence on how stakeholders can actually fulfil this change agent role as ambassadors. These findings suggest to involve stakeholders early in the implementation process and ensure clear communication about their roles and expectations. Additionally, we recommend to appoint project leaders and caregivers to facilitate this process by fostering support at all organizational levels.

According to the NASSS framework and CeHRes roadmap, close proximity and good cooperation are seen as fostering for implementation.^24,26^ Our study showed the close proximity of the project leaders’ with the care teams and other departments such as IT and management, which was experienced as helpful. Shared problem-solving allowed the care teams to learn from one another about using SCC. Problem-solving at the product level could be used to further develop the product during implementation.^23,25^ For example, the supplier of study has been working on an updated version of the product that addressed the issues also raised by the participants of this study. This illustrates the opportunity to further develop a product during implementation.

Our study also demonstrated that a shared vision and cooperation between the key stakeholders is important for incorporating new working routines, thereby ensuring adequate SCC use. This is in line with the constructs of the CFIR framework that should be taken into account when a (technological) innovation is being implemented.^15^ For example, the framework's constructs ‘teaming’, ‘planning’, and ‘tailoring strategies’ show the importance of working together in a team, aligning expectations, and adapting to the context. Careful consideration of these constructs can assist the appropriate and effective implementation of SCC use in care teams’ day and night work routines. Additionally, communication between stakeholders and across departments about the expected value of an innovation and interpersonal relations within an organization contribute to positive experiences and the perceived success of sustainable implementation. For example, the project leaders in our study kept close contact with care teams and departments such as IT and management by keeping them informed and engaged throughout the implementation and by ensuring communication about their roles and what was expected from them. Part of the project leaders’ and the caregivers’ motivation was found to be determined by the expected value of SCC for the people with PIMD and for them as caregivers. This is in line with research by Damschroder et al. (2009)^33^ and Greenhalgh et al. (2004)^17^ underlining the importance of intra-organizational communication and inter-organizational networks and how effective communication across these domains enhances the success of the implementation and routinization of new structures.^17^

Furthermore, our study showed the importance of skills and training and how this influenced the willingness to adopt SCC. The literature contends that the complexity of a technological innovation can hamper its adoption or at least lead to frustration in using the technological innovation adequately.^34^ Training is one way to overcome these difficulties – and some persons need less training than others – but it is as important to have a support network, i.e., caregivers helping one another to use SCC.^33^ In discussions with relevant stakeholders, the question of the potential added value of implementing the new technology should be raised with them. Doing so can foster the intention to change work routines by creating a common sense of the relative advantage, by planning carefully, and by involving key stakeholders.^16^

Our main findings suggest that shared problem-solving among care teams, ongoing training, and the development of a strong support network are essential to overcome challenges related to SCC. Finally, a shared vision between stakeholders of SCC's value will encourage the adoption and sustainability of new work routines. These recommendations can be used to inform and supplement the implementation guide used in this project.

## Limitations

This study is one of the first that addressed experiences with implementing SCC in disability care. We used an integrative framework combining the NASSS framework and the CeHRes roadmap to develop a broad topic list for data collection to obtain a broad perspective of the implementation process from the interviewees.^19^ Although the frameworks were not used in the analysis of the data, our findings provided insight into a number of the nine domains. For example, the domains participatory design/research, iterative process, value specification, and implementation were reflected in all themes, because the key stakeholders were actively involved and the implementation process was continuously evaluated to ensure that their needs and requirements were met. Having a shared vision gave substance to the domain value proposition, as it ensured that stakeholders knew what the added value was and fulfilled their role in this regard. The domain organization was elaborated on in all four themes; they showed which changes were necessary to implement and how these changes were achieved.

However, various other frameworks, theories, and models can be used to research and reflect on the implementation process for healthcare technologies such as technology acceptance model, unified theory of acceptance and use of technology, and CFIR.^14^ Using these theories might have highlighted different factors and components of healthcare technology implementation other than those in the NASSS framework and the CeHRes roadmap. Nevertheless, this study only used the integrated CeHRes roadmap and NASSS framework to collect the data structurally. We coded the data openly to leave room to identify other themes.

Four organizations and 15 interviewees participated in this study. The inclusion of all project leaders of these four organizations, combined with purposive sampling for caregivers, provided a broad range of experiences with the implementation process. Including different perspectives from other stakeholders, such as managers in the care organizations and the SCC suppliers, can capture additional important aspects of the implementation process.

## Conclusion

SCC implementation requires various stakeholders to be involved, contributing to the process to ensure use and make the implementation a success. Creating a common goal towards the expected value by ensuring early engagement and offering support are valuable and necessary steps. Moreover, the implementation of advanced care technologies in working routines requires efforts to communicate, coordinate, and motivate on the part of key stakeholders, such as caregivers, IT and logistics departments, and management. This study suggested that fostering communication between these key stakeholders and adequate problem-solving contribute to positive experiences and the success of sustainable implementation. This study offers a rich understanding of day-to-day practices around implementing SCC in disability care organizations – an understanding that may be of use for the implementation of technologies in other care settings too.

## Supplemental Material

sj-docx-1-dhj-10.1177_20552076241290399 - Supplemental material for The implementation of smart continence care for people with disabilities: A qualitative study of key stakeholders’ first-hand experiences Supplemental material, sj-docx-1-dhj-10.1177_20552076241290399 for The implementation of smart continence care for people with disabilities: A qualitative study of key stakeholders’ first-hand experiences by Julia FE van Calis, Vivette JC van Cooten, Odile Smeets, Jenneken Naaldenberg, Brigitte Boon, Geraline L Leusink and Kirsten E Bevelander in DIGITAL HEALTH

## Notes

a. Brand smart continence materials used in this study, Abena Nova, which was chosen for its market-readiness and CE-mark at the time of the grand proposal (2019).
